# Supplementary material for: Presence of male mitochondria in somatic tissues and their functional importance at the whole animal level in the marine bivalve Arctica islandica
Source: Commun Biol. 2021 Sep 20;4:1104. doi: 10.1038/s42003-021-02593-1 (PMC8452683; doi:10.1038/s42003-021-02593-1)
Supplement: Supplementary file 2 — Supplementary Information [file 42003_2021_2593_MOESM2_ESM.pdf]

Supplementary Figure 1. Distribution of ♀ and ♂ mtDNA in subarctic *Arctica islandica* populations.

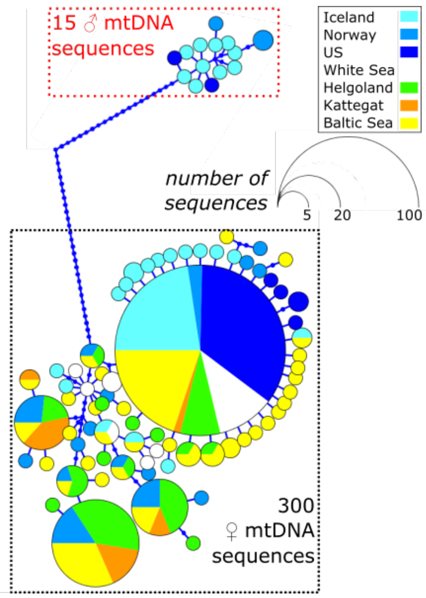

Mitochondrial haplotype network was built on concatenated cytochrome b and 16S partial sequences from *Arctica islandica* belonging to different populations. Branch-lengths correspond to the number of substitutions between haplotypes. The 127 bp indel in the 16S ♂ mtDNA partial sequence was replaced by a single segregating position in the alignment.

Supplementary Figure 2. Phylogenetic trees obtained from cytochrome b partial sequences haplotypes

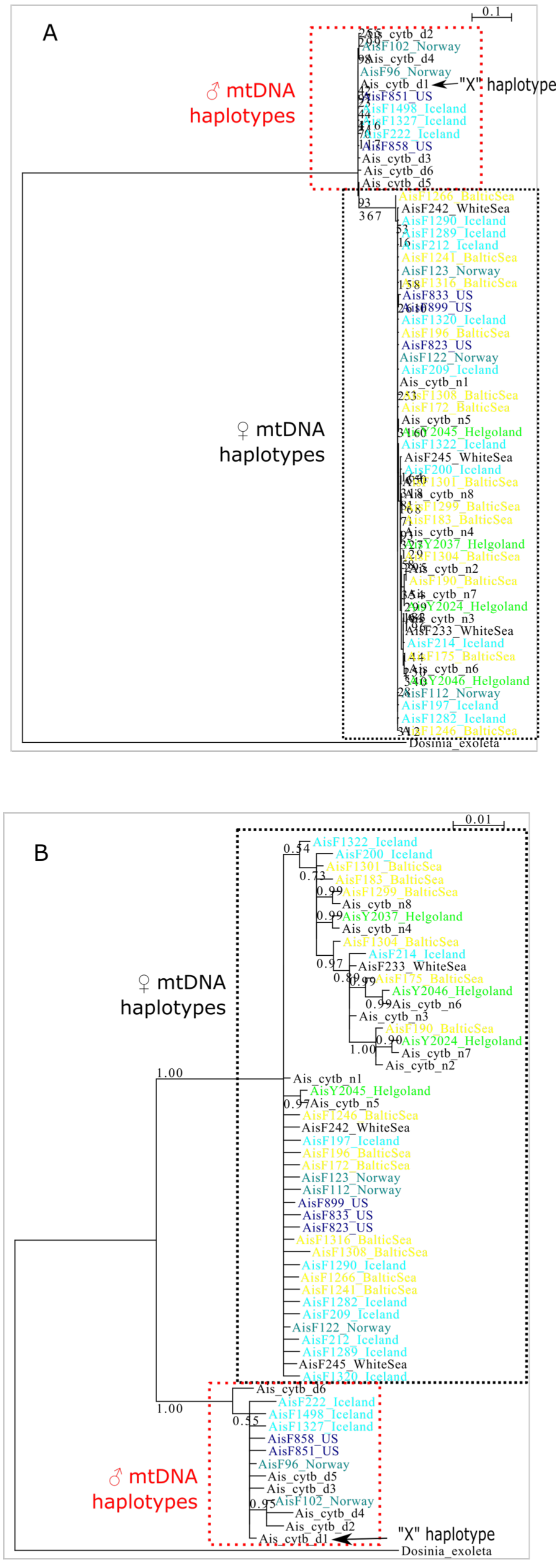

Maximum likelihood (PhyML) with bootstrap analysis (500 replicates) (A) and Bayesian phylogenies (MrBayes) (B) were used. Geographical origin of the sequence representing each haplotype are colored as in supplementary figure 1.

Supplementary Figure 3. Phylogenetic trees obtained from cytochrome b partial sequences haplotypes

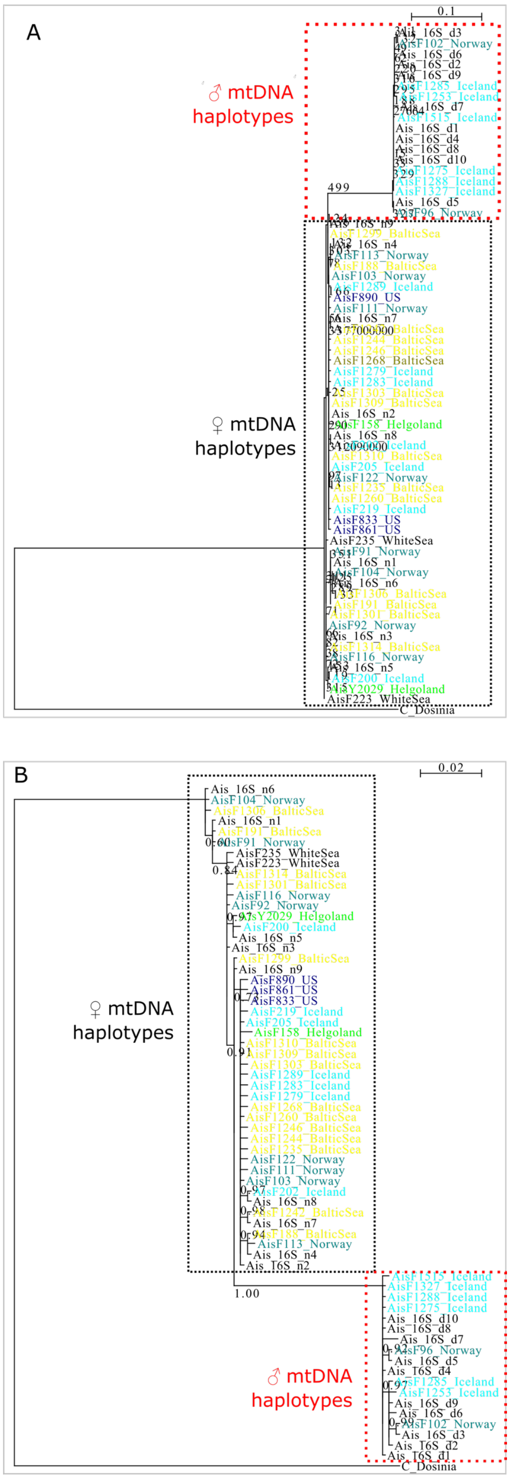

Maximum likelihood (PhyML) with bootstrap analysis (500 replicates) (A) and Bayesian phylogenies (MrBayes) (B) were used. Geographical origin of the sequence representing each haplotype are colored as in Fig S1.

Supplementary Figure 4. Representation of the standardized residuals between raw and predicted values for different morphological parameters.

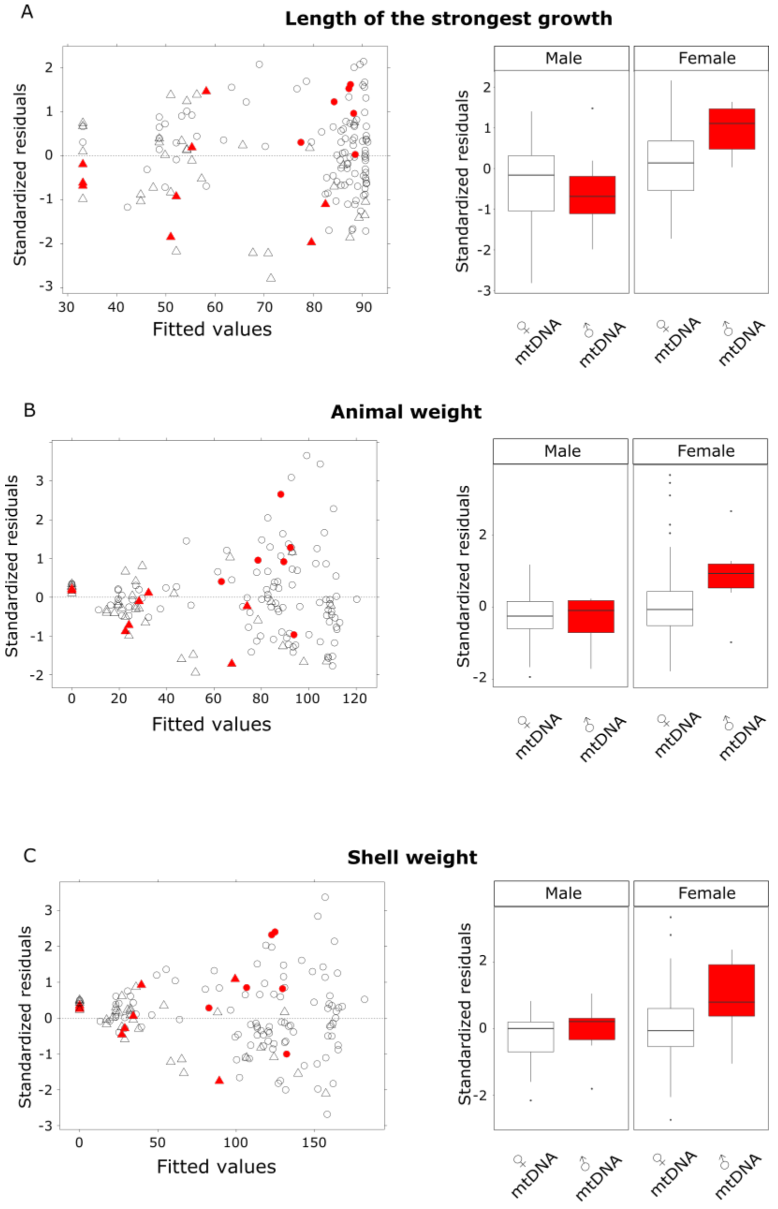

Length of the Strongest Growth (A), Animal Weight (B), Shell Weight (C) were used as morphological parameters. Left panels represent the standardized value over the predicted values and right panels represent the standardized value according to the sex and the mtDNA type of the individuals.

Supplementary Figure 5. Multiplex PCR test of a mock mixture of ♂- and ♀-type mtDNA simulating homo- and heterozygous conditions.

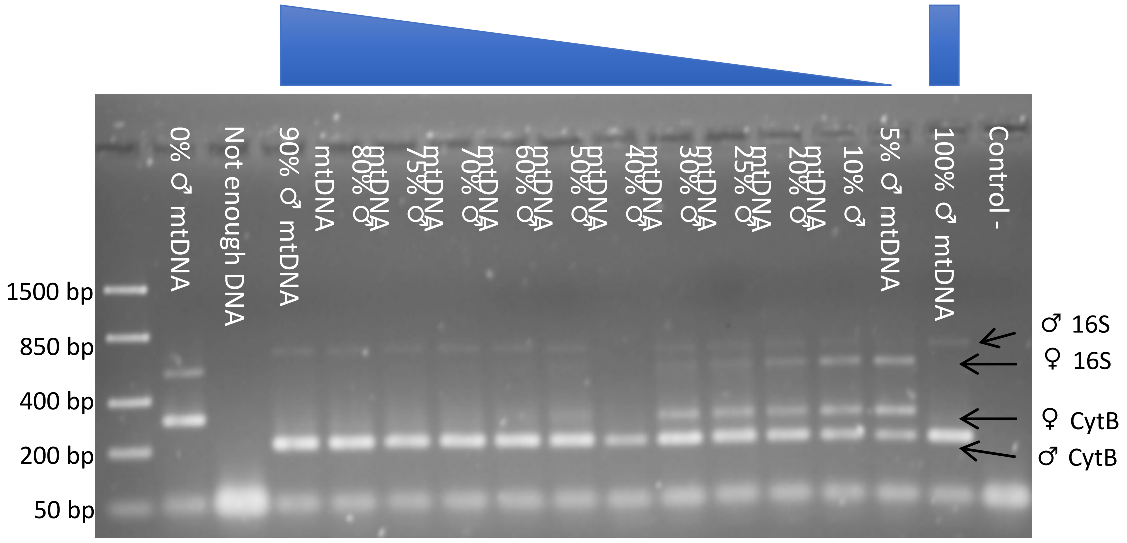

16S and CytB markers highlighting various size according to mtDNA type were used to ensure that the ♂-mtDNA is detected even when it only represents a low proportion of the total mtDNA mix. Percentage indicate the amount of ♂-mtDNA in the mix, the rest being completed with ♀ mtDNA.
