# Supplementary material for: Presence of male mitochondria in somatic tissues and their functional importance at the whole animal level in the marine bivalve Arctica islandica
Source: Commun Biol. 2021 Sep 20;4:1104. doi: 10.1038/s42003-021-02593-1 (PMC8452683; doi:10.1038/s42003-021-02593-1)
Supplement: Supplementary file 3 — Description of Additional Supplementary Files [file 42003_2021_2593_MOESM3_ESM.pdf]

Description of Additional Supplementary Files

File name: Supplementary Data 1

Description: 10.6084/m9.figshare.15002640
Comparison between F- and M- mtDNAs from Arctica islandica

File name: Supplementary Data 2

Description: 10.6084/m9.figshare.15006705
Primers and PCR conditions for amplifying 16S and cytochrome b from A. islandica

File name: Supplementary Data 3

Description: 16S sequences from all A. islandica population used for phylogenetic analysis

File name: Supplementary Data 4

Description: Cytochrome b sequences from all A. islandica population used for phylogenetic Analysis

File name: Supplementary Data 5

Description: 16S sequences from animals used for molecular investigations

File name: Supplementary Data 6

Description: Cytochrome b sequences from animals used for molecular investigations
