# Supplementary material for: Presence of male mitochondria in somatic tissues and their functional importance at the whole animal level in the marine bivalve Arctica islandica
Source: Commun Biol. 2021 Sep 20;4:1104. doi: 10.1038/s42003-021-02593-1 (PMC8452683; doi:10.1038/s42003-021-02593-1)
Supplement: Supplementary file 10 — Reporting Summary [file 42003_2021_2593_MOESM10_ESM.pdf]

nature portfolio

Corresponding author(s): Cyril Dégletagne

Last updated by author(s): Aug 3, 2021

Reporting Summary

Nature Portfolio wishes to improve the reproducibility of the work that we publish. This form provides structure for consistency and transparency in reporting. For further information on Nature Portfolio policies, see our Editorial Policies and the Editorial Policy Checklist.

Statistics

For all statistical analyses, confirm that the following items are present in the figure legend, table legend, main text, or Methods section.

n/a	Confirmed	
☐	☒	The exact sample size (*n*) for each experimental group/condition, given as a discrete number and unit of measurement
☐	☒	A statement on whether measurements were taken from distinct samples or whether the same sample was measured repeatedly
☐	☒	The statistical test(s) used AND whether they are one- or two-sided<br>*Only common tests should be described solely by name; describe more complex techniques in the Methods section.*
☐	☒	A description of all covariates tested
☐	☒	A description of any assumptions or corrections, such as tests of normality and adjustment for multiple comparisons
☐	☒	A full description of the statistical parameters including central tendency (e.g. means) or other basic estimates (e.g. regression coefficient) AND variation (e.g. standard deviation) or associated estimates of uncertainty (e.g. confidence intervals)
☐	☒	For null hypothesis testing, the test statistic (e.g. *F*, *t*, *r*) with confidence intervals, effect sizes, degrees of freedom and *P* value noted<br>*Give P values as exact values whenever suitable.*
☒	☐	For Bayesian analysis, information on the choice of priors and Markov chain Monte Carlo settings
☒	☐	For hierarchical and complex designs, identification of the appropriate level for tests and full reporting of outcomes
☒	☐	Estimates of effect sizes (e.g. Cohen's *d*, Pearson's *r*), indicating how they were calculated

Our web collection on statistics for biologists contains articles on many of the points above.

Software and code

Policy information about availability of computer code

Data collection	No software was used
Data analysis	All statistical tests were realized using R 3.2.1. All mitochondrial sequences were aligned within the Staden package to identify polymorphisms. MITOS online software (revision 917) was used to delimit mitochondrial protein coding genes, ribosomal and transfer RNA. Sequence differences (SNPs) between ♀ and ♂ mitochondrial genomes were counted in a sliding window of 500 with a step size of 1 and extracted using custom made software tools. These differences were considered if, for one given position, nucleotides from all ♂ mitochondrial DNA sequences are different from nucleotides from all ♀ mitochondrial DNA sequences, considering gaps as a fifth nucleotide. MEGA 6 (v6.06) was used to calculate p-distance between sequences (with bootstrap analysis 1000 replicates). Mitochondrial genome sequences are available on GenBank (accession numbers MG838904 to MG838921). To these canonical ♀ and ♂ mtDNA sequences the individual sequencing reads generated from the whole genome shotgun libraries were mapped using bowtie2. Regions characteristic for either ♀ or ♂ mtDNA were extracted and visualized with integrated genome browser . Cytochrome b and 16S sequences were aligned using the ClustalW algorithm of CodonCode Aligner program (v4.2.5, CodonCode Corporation, Dedham, MA, USA) and SeaView 57 (v4.5.4). Amino acid composition for cytochrome b was deduced using the invertebrate mtDNA genetic code (translation Table 5). MEGA 6 (v6.06)54 was used to calculate p-distance between sequences (with bootstrap analysis 1000 replicates). Maximum likelihood (PhyML) with bootstrap analysis (500 replicates) and Bayesian phylogenies (MrBayes) were performed on cytochrome b and 16S concatenated sequences with Dosinia exoleta (Bivalvia, Veneridae, Genbank accession number: cytochrome b - GQ166609.1; 16S – JF808184.1) as outgroup (closest sequence according to BLAST search) using Hasegawa-Kishino-Yano, with Invariable site and discrete Gamma distribution, nucleotide substitution model as determined by jModelTest2 58(BIC = 8905; lnL= 2351; version 1.6). The 127 bp indel occuring in the 16S partial sequences of individuals carrying the ♂ mtDNA was replaced by one nucleotide in this analysis and treated as a single event. FigTree (v1.4.4) was used to edit the phylogenetic tree. DNA haplotype networks were realized using Haplotype Viewer.<br>Twelve proteins encoded by the mitochondrial genomes of Veneroida species available (including ♀ mtDNA and ♂ mtDNA consensus mtDNAs) were aligned using the PRANK web server (https://www.ebi.ac.uk/goldman-srv/webprank/) , ambiguously aligned columns in the resulting alignments were identified and removed using BMGE v. 1.12_1 on the ngphylogeny server (https://ngphylogeny.fr) and a

concatenated alignment of 2697 amino acids was put together in Geneious Prime v2021.1.1. A Maximum Likelihood phylogenetic tree rooted with Crassostrea was calculated on the IQTree web server (http://iqtree.cibiv.univie.ac.at). The models of amino acid substitution best fitting to the data were estimated for each of the twelve partitions (representing protein coding genes) separately. The tree topology was tested for robustness by calculating the ultrafast bootstrap support in IQTree and the tree was edited in ITOL (https://itol.embl.de).

For manuscripts utilizing custom algorithms or software that are central to the research but not yet described in published literature, software must be made available to editors and reviewers. We strongly encourage code deposition in a community repository (e.g. GitHub). See the Nature Portfolio guidelines for submitting code & software for further information.

Data

Policy information about availability of data

All manuscripts must include a data availability statement. This statement should provide the following information, where applicable:

- Accession codes, unique identifiers, or web links for publicly available datasets
- A description of any restrictions on data availability
- For clinical datasets or third party data, please ensure that the statement adheres to our policy

Mitochondrial genome sequences are available on GenBank (accession numbers MG838904 to MG838921).
Sequencing data are attached to the paper as fasta files (Supplementary Data 1 -4)

Field-specific reporting

Please select the one below that is the best fit for your research. If you are not sure, read the appropriate sections before making your selection.

☒ Life sciences ☐ Behavioural & social sciences ☐ Ecological, evolutionary & environmental sciences

For a reference copy of the document with all sections, see nature.com/documents/nr-reporting-summary-flat.pdf

Life sciences study design

All studies must disclose on these points even when the disclosure is negative.

Sample size	For phylogeographic analysis, we used all A. islandica sampled. For qPCR and enzymatic measurements, we used all A. islandica with a male mitochondrial genome in their somatic tissue and around 20 individuals with the classical female mitochondrial genome (choose randomly with an equal number of male and female individuals)
Data exclusions	No data were excluded from the analysis
Replication	All qPCR tests and enzymatic measurements were realized in duplicates.
Randomization	A. islandica individuals were allocated to experiemental group according to their mitochondrial genotype and sex.
Blinding	Samples were designated by a number which did not contain any information regarding sample genotype or sex.

Reporting for specific materials, systems and methods

We require information from authors about some types of materials, experimental systems and methods used in many studies. Here, indicate whether each material, system or method listed is relevant to your study. If you are not sure if a list item applies to your research, read the appropriate section before selecting a response.

Materials & experimental systems

n/a	Involved in the study	
☒	☐	Antibodies
☒	☐	Eukaryotic cell lines
☒	☐	Palaeontology and archaeology
☐	☒	Animals and other organisms
☒	☐	Human research participants
☒	☐	Clinical data
☒	☐	Dual use research of concern

Methods

n/a	Involved in the study	
☒	☐	ChIP-seq
☒	☐	Flow cytometry
☒	☐	MRI-based neuroimaging

Animals and other organisms

Policy information about studies involving animals; ARRIVE guidelines recommended for reporting animal research

Laboratory animals	Not applicable
Wild animals	Frozen samples used in this study are from wild Arctica islandica and were collected from previously published studies.
Field-collected samples	As described in the article, before each experimentation, specimens were held in aquaria with recirculating water and course bottom sediment, to recover from sampling and transportation stress. Water parameters were adjusted to match the natural conditions at the collection site. Prior to dissection, animals were individually weighed, and the animal body weight was calculated by subtracting dry shell weight from whole animal weight. Different tissue were sampled, frozen in liquid nitrogen and stored at -80°C. Gonadic tissue were dehydrated, embedded in paraffin and histological sections were stained with haematoxylin/eosin solutions, observed under a Medicus HF:Hellfeld Microscope (Hund, Wetzlar, Germany) at 10x magnification to determine A. islandica sexes. Shell line of strongest growth (LSG) were measured with calipers to the nearest mm. Individual age (up to 226 years old) was deduced from shell growth bands as previously described.
Ethics oversight	No ethical approval was required as we are studying Arctica islandica, a commercially used, abundant and non endangered marine invertebrate. Moreover, we would like to state here that all tissues used in this study were frozen from previous studies. No animals were taken from the environment specifically for the present study.

Note that full information on the approval of the study protocol must also be provided in the manuscript.
